# Pattern of Disinfection of Root Canal Dentin by Alternated Acid-Base Irrigating Regimen

**DOI:** 10.1155/2019/9219238

**Published:** 2019-01-01

**Authors:** Janir Alves Soares, Maria Auxiliadora Roque de Carvalho, Suelleng Maria Cunha Santos Soares, Rodrigo Dantas Pereira, Manoel Brito-Júnior, Paula Prazeres Magalhães, Isabel Alessandra Miranda Nunes, Rudys Rodolpho de Jesus Tavarez, Claudia de Castro Rizzi, Rafaela Nogueira Moreira, Luiz de Macêdo Farias, Etevaldo Matos Maia Filho

**Affiliations:** ^1^Department of Dentistry, Federal University of the Jequitinhonha and Mucuri Valleys, Diamantina, Minas Gerais, Brazil; ^2^Department of Microbiology, Institute of Biological Sciences, Federal University of Minas Gerais, Belo Horizonte, Minas Gerais, Brazil; ^3^Department of Dentistry, State University of Montes Claros, Montes Claros, Minas Gerais, Brazil; ^4^Department of Dentistry, Ceuma University, São Luís, Maranhão, Brazil

## Abstract

**Objective:**

To quantify* Enterococcus faecalis* density in root canal dentin after chemomechanical preparation (CMP) using alternated irrigating regimen.

**Methodology:**

Root canals (RC) were contaminated with* E. faecalis* (ATCC 19433) for 3 weeks and evident biofilms were obtained. After initial sampling (S1), the CMP was aided by irrigants: saline solution (control; n=12), a conventional regimen (CR) (group 1; n=12) using 5.25% NaOCl and a final rinse with 17% EDTA, and an alternating regimen (AR) of intercalated use of NaOCl and EDTA (group 2, n=12), followed by a second sampling (S2). After 2 weeks, S3 was obtained. Two roots were analyzed by scanning electron microscopy. Each root was divided into cervical, mild, and apical segments and sampling of the superficial (n=90) and deep (n=90) dentin layers was obtained using Gates-Glidden burs. The* E. faecalis* density (CFU/mg) in log_10_ was categorized as residual (0 > 0.2), moderate (0.2 ≥ 0.5), or elevated (> 0.5). The prevalence of positive samples in BHI and BHI-A was analyzed by Pearson's chi-square test. The data were normalized by a log_10_ transformation of CFU and were analyzed by one-way ANOVA and Tukey's tests.

**Results:**

Biofilms were observed only in the control root canal walls. Topographically, the controls and CR showed similar distributions of* E. faecalis* in the dentin. Microbiologically positive root canals harbored much* E. faecalis* in the adjacent dentin (*p* < 0.05). Irrigating saline provided moderate density of* E. faecalis *in the dentin while CR and AR resulted in a residual density of microorganisms (*p* < 0.05).

**Conclusions:**

The* Enterococcus faecalis* density in dentin was influenced by the irrigating regimen and the microbiological status of the root canal. The CMP aided by the alternating regimen interfered with the recolonization of the root canal and topographic distribution of* Enterococcus* in root dentin.

## 1. Introduction

In the teeth with pulp necrosis the spaces of the pulp cavity become unprotected by the immune system and this makes this environment favorable to the proliferation of various microorganisms [1]. Such microorganisms form biofilms on the walls of the root canals and colonize the adjacent dentin structure [2–4] and are associated with the formation and persistence of periapical lesions [3, 4]. Clinically, from a therapeutic point of view, an important focus is oriented to achieve negative microbiological culture prior to root canal [5–7] and the efficacy of antisepsis protocols can be routinely assessed through the microbiological culture and confocal laser scanning microscopy [2].

Then, from a therapeutic point of view, an important focus is oriented to achieve negative microbiological culture prior to RCS obturation [6–8]. The chemomechanical preparation has an unquestionable antisepsis power, but with effectiveness concentrated to the main root canal [4, 9]. Several protocols with the placement of an interappointment intracanal dressing are required, but the elimination of the remaining primary infection in the RCS is challenging [10, 11]. Furthermore, in root canal treatment failures, anatomical factors and operative technical errors [12] add to the microbiological aspect [7, 13], with high prevalence of* Enterococcus faecalis* [14]. These bacteria form biofilms [15, 16], present resistance to some antiseptic agents [15, 17], and colonize the dentin tubules [16, 18]. In these circumstances, the dentin acts as a reservoir for the microorganisms [19, 20], which should be the most reasonable explanation for the resurgence of the apical periodontitis lesion [12, 21].

Traditionally, the antisepsis protocols' effectiveness is evaluated only on the root canal level and in a short time interval after the procedures [4, 11, 22, 23]. Thus, monitoring their longevity in an extended period, as well as evaluating the microbiological condition of the adjacent dentin in models contaminated with resistant microorganisms, would be an important challenge for their validation. Therefore, the aim of this study was to quantify* E. faecalis* in the root dentin in superficial and deep extensions two weeks after chemomechanical preparation using different irrigation regimes. Additionally, the dentin contamination level was compared with the microbiological state of the main root canal.

## 2. Materials and Methods

### 2.1. Tooth Selection and Root Canal Contamination

After clinical and radiographic evaluation, 36 human canines with similar root canal morphology were selected. The teeth were donated by a university tooth bank. The root mean length was 16 mm, the root canal trajectory was radiographically visible, the degree of curvature was less than 25°, and the thickness of the mesial and distal dentin walls was superior to 1.5 mm in the two coronal thirds. The crowns were removed using diamond discs at low speed under sterilized saline solution irrigation (KG Sorensen, São Paulo, Brazil), obtaining apical patency with a #15 K-file (Maillefer Instruments SA, Ballaigues, Switzerland) and the apex was prepared to a #25 K-file. The root canals were irrigated with 5.25% NaOCl followed by a final rinse with 17% EDTA (Lenza Farmacêutica Ltda, Belo Horizonte, MG, Brazil) for smear layer removal. Each root was autoclaved and the apical foramen was sealed with a cyanoacrylate in a laminar flow chamber (Marconi Equipment, Piracicaba, São Paulo, Brazil). The root canals were inoculated with a fresh suspension of* E. faecalis* (ATCC 19433) at 10^9^ CFU/mL in BHI, followed by incubation at 37°C, according to Soares et al. [24]. Every 48 hours, the root canals were reinoculated with fresh suspension until a final period of 3 weeks.

### 2.2. Intracanal Antisepsis

Prior to chemomechanical preparation, the initial microbiological sample (S1) was taken with three #25 sterile absorbent paper points (Tanari Ind. Ltda, Manacapuru, AM, Brazil) which after remaining in the root canal for 1 minute were transferred to Eppendorf tubes with 1000 *μ*L of saline solution and the microbiological processing was completed. Established protocols were adopted. The root canals were manually prepared by the crown-down technique using K-files and Gates-Glidden burs (Dentsply Maillefer). After coronal flaring with Gates-Gliddens, the apical enlargement was extended to a #40 K-file following the step-back technique and irrigation using an up-and-down motion was performed at every change of the file. Each root canal was irrigated with 15 mL of solution, with 1 mL of irrigant dispensed after each instrument. In the control group (n=12), the canals were irrigated with 0.85% saline solution. In the group treated according to the conventional regime (group 1, CR, n=12), 13 mL of 5.25% NaOCl was used, followed by 1 mL of 17% EDTA, for 3 minutes, aided by the master apical K-file #40 for smear layer removal, and new irrigation with 1 mL of NaOCl. In the group irrigated by the alternating regimen (group 2, AR, n=12) during the instrumentation, 14 mL of 5.25% NaOCl was used with the alternating application of 17% EDTA in a total of 1 mL. The CR and AR groups differed from each other only by the way the EDTA solution was used. In the CR group, the entire EDTA volume was dispensed at same time, and, in the AR group, the 200 *μ*L EDTA aliquots were applied in an intercalated manner after NaOCl irrigation. After the CMP, the RC was irrigated with 2 mL of 0.85% saline. To obtain the second microbiological sample (S2) with three #40 sterile absorbent paper points, the microbiological samples were processed and the root was incubated at 37°C. After 2 weeks, the sample S3 was obtained as previously described. In each group, two roots were randomly selected, and, after being longitudinally sectioned, the samples were dehydrated in increasing order of alcohol from 50% to 100%, mounted on stubs, gold-sputtered, and evaluated under a SEM (JEOL-JSM 5600LV, NORAN Instruments, Tokyo, Japan) at 15 kW. Biofilm formation, isolated* E. faecalis*, and patent dentin tubules were evaluated in 10 sites in each third of the root canals, using magnifications ranging from 1000 to 10000X.

### 2.3. Dentin Samples

**Figure 1:**
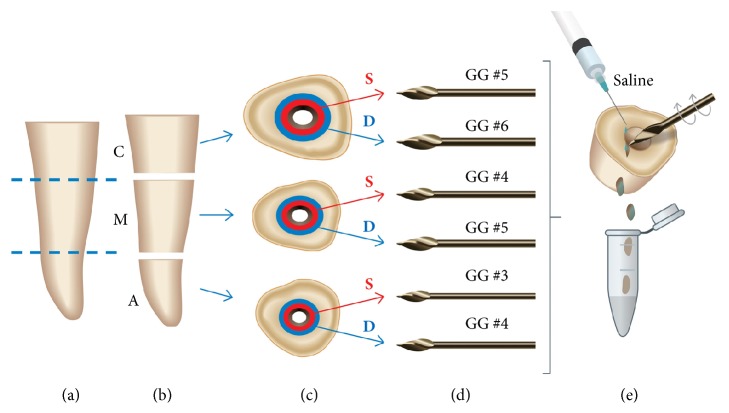
Schematic drawing of the dentin samples collection. (a) Root of human canine tooth. (b) Root sectioned into coronal (C), middle (M), and apical (A) segments. (c) Cross section of root segments showing the regions of the superficial (S) and deep (D) dentin. (d) Gates-Glidden (GG) drills #3 to #6 were used for obtaining dentin chips. (e) Dentin chips collected in microtubes under irrigation with sterilized saline.

According to illustrative Figure 1, the roots of each group were transversely sectioned in the cervical (n=10), middle (n=10), and apical (n=10) segments using diamond discs at low speed under sterilized saline solution irrigation. From each segment, microbiological samples of the dentin structure in two extensions were taken using two Gates-Glidden (GG) burs of progressive diameters. The dentin adjacent to the root canal constituted the superficial sample and the dentin that extended to the proximities of the cementum represented the deep sample. In the cervical, middle, and apical segments, GG #5/#6, #4/#5, and #3/#4 were used, respectively. The superficial and deep samples were obtained with smaller and greater GG diameters, respectively. During the GG action, the lumen of the root canal was constantly irrigated with 1000 *μ*L of saline solution. The burs were circumferentially oriented through root canal walls and the dentin chips were directly collected in Eppendorf tubes. The weight of the superficial and deep dentin was indirectly determined. The 90 cylinders with approximately 5 mm of extension were individually weighed in an electronic analytical balance (Gehaka, Industria e Comércio Ltda, São Paulo, SP, Brazil) and the initial weight (W1) was registered. After taking superficial samples, the segments were dried with paper cones and sterilized gauze and weighed again (W2). The difference W1-W2 determined the S weight. Similarly, the deep samples and W3 were obtained. The difference W2-W3 determined the deep dentin weight. The 180 dentin samples were microbiologically processed. The density of microorganisms on dentin (CFU/mg) was considered to be residual (0 > 10), moderate (10 ≥ 100), and elevated (> 100), and the equivalent log_10_ scale was 0 > 0.2, 0.2 ≥ 0.5, and > 0.5, respectively.

### 2.4. Microbiological Processing

For microbiological processing, the samples were vortexed for 30 seconds. Tenfold dilutions were prepared and 100 *μ*L aliquots of the suspension were spread onto BHI-agar media (BHI-A; Difco, Detroit, MI) in triplicate and incubated at 37°C for 72 hours. Colony-forming units per mL (CFU/mL) were enumerated. The remainder was added in BHI broth (BHI, Difco) and incubated at 37°C. Monoinfection by* E. faecalis* was confirmed by Gram staining and catalase testing.

### 2.5. Statistical Methods

The prevalence of positive samples in BHI and BHI-A was analyzed by Pearson's chi-square test. The data were normalized by a log_10_ transformation of CFU and were analyzed by one-way ANOVA and Tukey's tests. Values of* p* < 0.05 were considered significant.

## 3. Results

**Figure 2:**
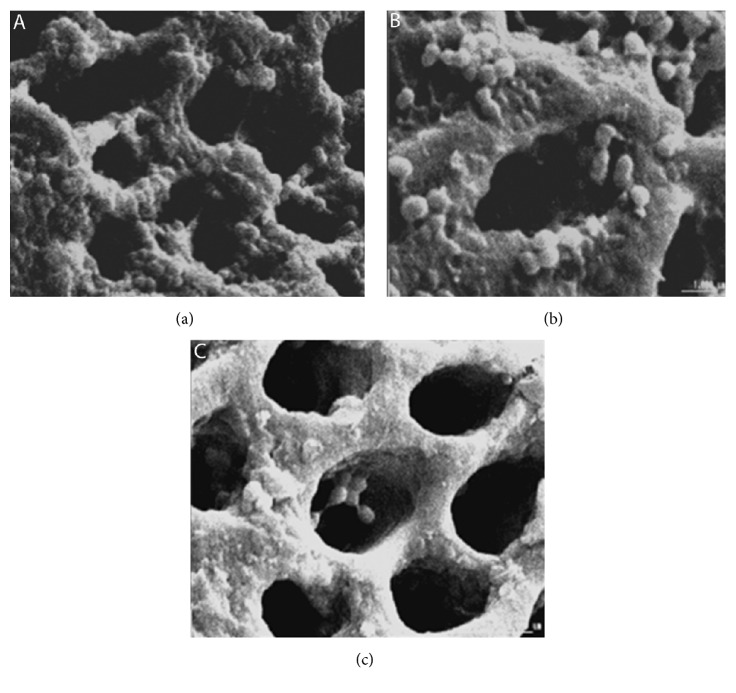
SEM micrographs of root canal walls and dentinal tubules. Control group: (a)* E. faecalis *biofilms on dentinal walls. Conventional regimen: (b) scattered* E. faecalis* in the root canal wall and dentin tubules entrance. Alternating regimen: (c) scarce* E. faecalis* in entrance dentin tubules. Magnifications: (a) 5000X, (b) 7200X, and (c) 10.000X.

In the initial microbiological sample all the root canals provided quantitative* E. faecalis* growth. After chemomechanical preparation the CFU average reduction was similar between CR and AR irrigation (*p* > 0.05), which were significantly superior to the control (*p* < 0.05). After 14 days the values of 1.3 × 10^3^ (range: 0.8 × 10^3^ to 1.5 × 10^3^), 2.2 × 10^2^ (range: 1 × 10^2^ to 2.4 × 10^2^), and 0 CFU for the control, CR, and AR, respectively, were recovered. In this last phase, a similar microbiological condition was verified between controls and CR (*p* > 0.05). Using SEM, biofilms were observed only in the root canal of the control (Figure 2(a)). In CR and AR, scattered* E. faecalis* were in the root canal wall (Figure 2(b)) and dentinal tubules (Figure 2(c)).

**Figure 3:**
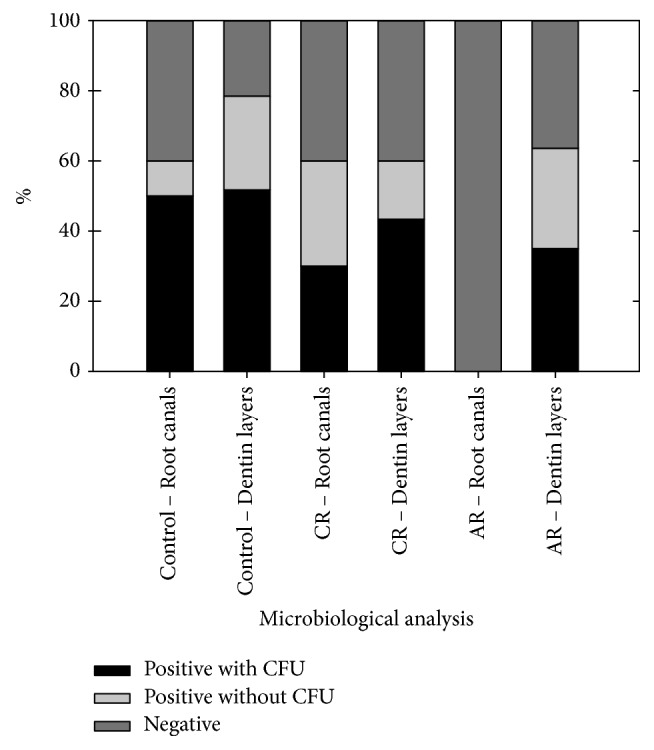
Relative frequency of the microbiological conditions in the root canals and respective dentin. Control group: irrigation with 0.85% saline; group 1: irrigation with NaOCl, final rinse with EDTA, and irrigation with NaOCl (CR); and group 2: irrigation with a NaOCl/EDTA/NaOCl combination (AR).

Figure 3 summarizes the relative frequency of samples positive for* E. faecalis* in the root canals and respective dentin. These results showed that the control and CR presented intracanal microbiological conditions that were significantly different from AR (*p* < 0.05), but all groups presented similar prevalence of microbiologically positive dentin layers (*p* > 0.05).

**Figure 4:**
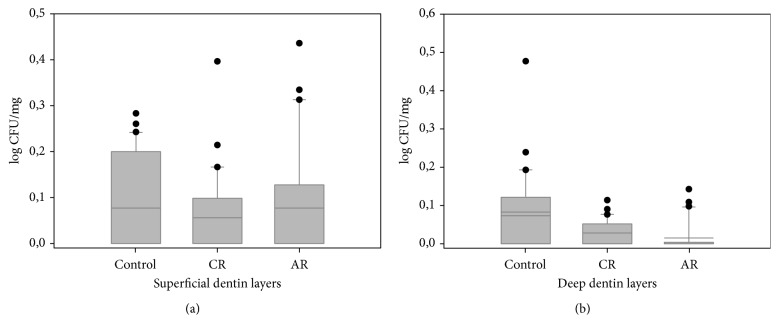
Box plot of* E. faecalis* amount (log⁡CFU/mg) in the superficial (a) and deep (b) dentin layers.

In the microbiological analysis density in the control, CR, and AR was of the order of 93.8, 9.7, and 3.1 CFU/mg of dentin, respectively, and the results were grouped as a function of superficial and deep dentin layers (Figure 4). The means for CR and AR were similar (*p* = 0.81), but both were significantly inferior to the control (*p* < 0.05). The density of* E. faecalis* was significantly greater in superficial than in deep dentin only in AR (*p* < 0.05).* E. faecalis* showed similar density in the superficial layers of the three groups (*p* > 0.05) (Figure 4(a)). The control presented significantly more* E. faecalis *in the deep dentin layer than CR and AR (*p* < 0.05) (Figure 4(b)).

According to Table 1, it was verified that all root canals of the control and those microbiologically positive samples of CR presented density of* E. faecalis* varying from moderate to elevated (*p* < 0.05). The microbiologically negative root canals of the CR and all samples of AR harbored residual density of* E. faecalis* (*p* > 0.05).

## 4. Discussion

In experiments with dentin samples, the root canals were contaminated for one [25], three [26], or seven [27] days. We chose 3 weeks because* E. faecalis* can achieve 800-1000 *μ*m in the dentin tubules in this period [18] and old* E. faecalis* biofilms were obtained in the root canal [16, 28]. It seems clear that, in more than half of the infected roots, bacteria are present in the deep dentin close to the cementum [29]. In human pulpless teeth, in an extension of 0.5 to 2.0 mm, 67.7% of the samples presented microorganisms [19] at a density of 10^2^ CFU/mg at 5 × 10^4^ CFU/mg of the dentin [30].

In our study, in terms of procedures for intracanal antisepsis, manual instrumentation was used associated with three irrigation regimens of the root canal. In the current state of the art the irrigation regimens in vitro employing EndoVac, PUI, sonic irrigation, and XP-Endo Finisher have provided superior performance comparatively to a manual irrigation regimen [31]; however, the level of scientific evidence is still fragile [32].

In this study, the chemomechanical preparation provided logarithmic reduction of* E. faecalis* in the root canal. According to Figdor et al. [33], cell density may remain stable despite a long period of nutrient depletion. However, in clinical practice, microorganisms may be recovered in concentrations similar to those detected before endodontic therapy during a period of 48 to 72 hours after CMP [6, 32]. In the medium term, it was demonstrated that the irrigation regimen influenced root canal recolonization and the microbiological standard of the dentin. Therefore, comparatively to the saline, the irrigation alternating regimens based on NaOCl and EDTA promoted greater CFU reduction (*p* < 0.05). Through microbiological monitoring, the control group presented similar microbiological aspects in the S2 and S3 steps. Certainly, a numeric increase of* E. faecalis* required intracanal nutrient availability [31], but this did not happen. However, opposing microbiological changes in the root canals irrigated with NaOCl and EDTA were verified as a function of the simple irrigation regimen. Thus, under similar nutritional depression, the root canal recolonization irrigated by the conventional regimen reestablished a microbiological scenario similar to the control. However, through the alternated irrigation regimen, the root canals became completely free from microorganisms. In this context, microbiological evaluations in the adjacent dentin were performed.

It was verified that the* E. faecalis* density was influenced by the irrigation regimen, by the specific microbiological conditions in the root canal, and, in a shorter extension, by the dentin depth. It was also noted that the microbiologically positive root canals presented significantly greater* E. faecalis* density in the dentin compared to the negative ones. In this way, in the control, 155 and 12.3 UFC/mg of dentin were recovered from the positive and negative canals, respectively. For the group irrigated with the conventional regimen, these values were 15.7 and 0.2, respectively (*p* < 0.05). In terms of segments, the root morphology determined a significantly shorter dentin mass in the apical third, but without a significant difference in the* E. faecalis* density among the three segments. In general, the mass D was practically the double of S; however, the* E. faeca*lis density was similar between the layers of the control and conventional regimen groups. We also checked that* E. faecalis* was recovered in an extensive dentin thickness, much more than that recommended by the root canal instrumentation techniques. Thus, it would be unjustifiable to excessively increase the root canal enlargement to reach its mechanical elimination. This means that the preparation with an instrumentation technique removing substantial amounts of dentin did not reduce microorganisms more effectively from the root canal system than a more conservative instrumentation technique [33].

Johal et al. [34] showed consistent elimination of* E. faecalis* in root canals with 5.25% NaOCl and 15% EDTA in vitro. Moreover, in clinical retreatment, microorganisms could be detected in none of the teeth following proper root canal preparation with 2.5% NaOCl and 17% EDTA [35]. Ozdemir et al. [36] demonstrated that the combination of EDTA and NaOCl significantly reduced the amount of intracanal biofilms of* E. faecalis*. In a previous study, Soares et al. [23] obtained 100% germ-free root canals using an alternative regimen based on NaOCl and EDTA. Using this model, the study demonstrated that negative microbiological conditions at the root canal level can present inverse microbiological aspects in the adjacent dentin. Moreover, the density and topographic disposition of* E. faecalis* was influenced by the intracanal irrigation regimen. Thus, positive canals irrigated with saline had* E. faecalis* density in the dentin that was significantly greater than those irrigated with the regimens based on NaOCl and EDTA. It was also observed that none of the microbiologically negative root canals were germ-free in all the dentin layers.

Worldwide, the combination of sodium hypochlorite (NaOCl) and EDTA has been used for antisepsis of root canal systems. NaOCl has a broad antimicrobial spectrum and dissolves organic matter [37]; EDTA is a chelating agent that aids in smear layer removal [38] and increases dentin permeability [39]. The alternated irrigation regimen provided three times less* E. faecalis* (3.2 UFC/mg) compared to the conventional regime. Surprisingly,* E. faecalis* was mainly concentrated in the superficial dentin layer, and, paradoxically, no root canal was recolonized. Besides eliminating most microorganisms in the root canal systems, this alternating regimen can hypothetically modify the dentin surface, hindering the adhesion and later the recolonization of the root canal wall by* E. faecalis*. It has been reported that EDTA reduces the hydrophobicity and surface free energy of root dentin [36] and thereby influences the nature of bacterial adhesion, adhesion forces, and biofilm formation of* E. faecalis* to dentin [40]. Exposure to EDTA (50 mmol/L) affected the membrane integrity of* E. faecalis* [41]. NaOCl is an efficient organic solvent that causes dentin degeneration because of the dissolution of collagen by breaking down bonds between carbon atoms and disorganizing the proteic primary structure [42]. 5.25% NaOCl associated with 17% EDTA resulted in a loss of dentin structure to provoke alterations in the dentin collagen, resulting in disorganized patterns near the root canal [43]. An alternating regimen using citric acid and NaOCl 1% optimized the elimination of* E. faecalis*, possibly due to the increase in dentinal permeability [44, 45]. Although the exact mechanisms of EDTA and NaOCl interaction on dentin and* E. faecalis* biofilms are not yet known, their antiseptic efficacy deserves more complementary studies [45, 46].

Similarly to Mickel et al. [47], we also verified bacteria on dentinal walls and in tubules by SEM, even in negative canal cultures. Therefore, concern exists as to the fate and clinical implications of the remaining microorganisms in the root canal system [10, 13]. In fact, an intracanal negative culture is still the best indication of the maximum reduction of infection at a compatible level with periradicular tissue healing [22]. Therefore, it would be interesting to investigate the complementary antisepsis strategies to eliminate such* E. faecalis* located in this superficial dentin as an example of photodynamic therapy, iodine-potassium iodide or calcium hydroxide [31, 48]. Further works should attempt to develop effective protocols for the antisepsis of root canal systems in one session.

In conclusion, at an average interval of time under conditions of starvation, after biomechanical preparation using different irrigation regimes, the density of* E. faecalis* in dentin ranged from residual to elevated. The alternated irrigation regimen with NaOCl and EDTA provided superior elimination of* Enterococcus faecalis* in root canal and adjacent dentin.

## Figures and Tables

**Table 1:** Intracanal microbiological conditions and *E. faecalis* density in the adjacent dentin in log⁡scale.

Groups	Root canal culture	log⁡CFU/mg^*∗*^	Equivalent density of *E. faecalis*
Control (n=10)	Positive (n=6)	0.97 a	elevated
	Negative (n=4)	0.37 b	moderate

Conventional regimen (n=10)	Positive (n=6)	0.44 b	moderate
	Negative (n=4)	0.01 c	residual

Alternating regimen (n=10)	Negative (n=10)	0.17 c	residual

*∗*: different letters show statistically significant differences (one-way ANOVA, Tukey's test, *p* < 0.05).

## Data Availability

The data used to support the findings of this study are available from the corresponding author upon request.
